# Social inequalities in life expectancy and mortality during the transition period of economic crisis (1993–2010) in Korea

**DOI:** 10.1186/1475-9276-11-71

**Published:** 2012-11-21

**Authors:** Mia Son, Youngtae Cho, Juhwan Oh, Ichiro Kawachi, Junhyeok Yi, Soonman Kwon

**Affiliations:** 1 Department of Preventive Medicine, Medical College, Kangwon National University, Kangwon, South Korea; 2 School of Public Health, Seoul National University, Seoul, South Korea; 3 Institute of Health Policy and Management, Medical Research Center, Seoul National University, Seoul, South Korea; 4 Department of Society, Human Development, and Health, Harvard School of Public Health, 677 Huntington Avenue, Boston, Massachusetts, 02115, USA; 5 Korea Institute of Oriental Medicine, Daejeon, Korea

**Keywords:** Social inequalities, Life expectancy, Mortality, Economic crisis, Korea inequality in health

## Abstract

**Backgrounds:**

This study examines social inequalities in life expectancy and mortality during the transition period of the Korean economic crisis (1993–2010) among Korean adults aged 40 and over.

**Methods:**

Data from the census and the national death file from the Statistics Korea are employed to calculate life expectancy and age-specific-death-rates (ASDR) by age, gender, and educational attainment for five years: 1993, 1995, 2000, 2005, and 2010. Absolute and relative differences in life expectancy and Age-Specific Death Rates by educational attainment were utilized as proxy measures of social inequality.

**Results:**

Clear educational gradient of life expectancy was observed at age 40 by both sexes and across five time periods (1993, 1995, 2000, 2005, and 2010). The gradient became notably worse in females between 1993 and 2010 compared to the trend in males. The educational gradient was also found for ASDR in all five years, but it was more pronounced in working age groups (40s and 50s) than in elderly groups. The relative disadvantage of ASDR among working age Korean adults, both males and females, became substantially worse over time.

**Conclusions:**

Social inequalities in life expectancy and ASDR of the working age group across socioeconomic status over time were closely related to the widening of the social difference created by the macroeconomic crisis and the expansion of neo-liberalism in Korea.

## Introduction

Republic of Korea (Korea hereafter) experienced harsh economic crisis due to failure of foreign currency management in the late 1990s. To overcome the crisis, the Korean Government decided to receive special economic relief loan (US$21 billion) from the International Monetary Fund (IMF). Although they repaid 90% of the loan to IMF and officially announced the end of crisis in 2001, the toll of overcoming economic crisis was to accept a comprehensive structural adjustment program requested by IMF. This program required a financial and corporate restructuring, a tighter but market-oriented macroeconomic policy, and an increase of labor market flexibility, all of which are largely based on the "neo-liberal" ideology [1,2]. Under the restructuring program, not only the economic sectors but also almost all spheres of Korean society, including culture and value systems, have changed. Shortly after the break of foreign currency crisis, the unemployment rate soared to 8.6% in February 1999 from 2.2% in July 1997 [3]. As the labor market became flexible, the customary lifetime-employment policy disappeared and a huge number of workers aged mid 40s to early 50s, mostly those of low socioeconomic status, had to leave their work, experiencing downward social mobility. The sudden surge of unemployment rate resulted in massive family dissolution among the mid-aged and the delay or avoidance of marriage and childbearing among the young adult. Further, the *Gini* coefficient increased from 0.283 in 1997 to 0.337 in 2006, indicating aggravated socioeconomic polarization in Korean society [4,5].

Recently, a growing number of studies document that the expansion of neo-liberal economic environment is closely related to the widening social stratification in health and mortality. Although opposite evidence was found in Scandinavian countries in the early 1990s [6,7], it has been observed that health and mortality inequality becomes aggravated when neo-liberal market oriented economic system expands in a country [8,9]. Two causal mechanisms between neo-liberalism and widening health inequality can be considered. First, general consequences of the introduction of neo-liberalism are socioeconomic polarization and increased poverty among the lower class. As labor market becomes more flexible and more focused on the interest of employer, workers with less skills and knowledge often become the subject of downward social mobility or unemployment. Unless there is a social safety net system that buffers the adverse impact of labor market insecurity, material deficiency as well as the feeling of social exclusion increase among the poor. These processes naturally result in the degradation (or at least no promotion) of their physical and mental health, widening the health gap relative to those less affected by insecure labor market conditions. Second, commodification of public goods that used to be protected by the government is one of the major characteristics of neo-liberalism. Medical care systems and organizations are typical examples of public goods. Inception of market interest in health-related industry resulted in the escalation of medical service price, which in turn functions as a limitation to the access and utilization of medical services particularly for those with little socioeconomic resources, enlarging social inequality in health.

Given Korea's experience of economic crisis and the spread of neo-liberal market oriented economy, it is very likely that the social gap in mortality became worse after the crisis. Although there have been several studies that examined the existence of social disparity in health/mortality in the 2000s, studies concerning both pre- and post-economic crisis (or transitional) periods and the changes of social inequalities in mortality are scarce in Asia including Korea. The purpose of this study is therefore to empirically examine to what extent social disparity in mortality have aggravated in Korea since the economic crisis or the introduction of neo-liberal market economy. We focus only on mortality here, since death is generally the consequence of ill health, and therefore a worsening mortality gap across social groups, if any, within a relatively short time period (17 years) may suggest worsening health inequality.

## Methods

### Data

We used two national data sources, the Korean national death registry files and the Korean census data in 1993, 1995, 2000, 2005, and 2010, to calculate educational inequalities in life expectancy and age-specific death rates (ASDR) [10]. The reason that 1993 was considered here, although census was taken in 1990, is that the Korean national death registry file began to classify educational attainment from 1993. Age-specific population size by educational attainment in 1993 was the average of corresponding populations between 1990 and 1995. We divided our data into three time points: before (1993–1995), during (2000), and after (2005–2010) the economic crisis to observe how social inequalities in mortality changed over time. This study used educational attainment as a proxy for social class because of the following two reasons: 1) several previous studies in Korea show that the level of education mirrors social class more clearly than any other proxy indicators [11], and 2) educational attainment is the only available information in the Korean death files regarding socioeconomic status. Education is classified into four categories: elementary school or less (0–6 years), middle school (7–9 years), high school (10–12 years), and college or up (> = 13 years).

Adults aged 40 and over are considered in the current study due to the following two reasons, even though younger age groups are vulnerable to economic crisis as well. First, young adults aged 20s to 30s are physiologically healthy population with very low death rates, regardless of their socioeconomic status. Since health is a cumulative construct, it is likely that the adverse impact of low socioeconomic status during the 20s and the 30s on health, if any, may result in immature death after they turn to at least 40 years old. Second, education, the focal measurement of socioeconomic status in this study, is unlikely to be completed yet until the mid to late 30s in age in Korea.

### Analysis

We constructed 5-year abridged life tables based on education and sex specific ASDRs from age 40 for the three time points. Life expectancy was calculated using the *Survival* program (version 7.0) with an option to estimate the age-specific survival probability based on the Keyfitz-Frauenthal formula [12]. Life expectancies at age 40 of four educational attainment subgroups over five time points were calculated and compared to examine the extent of baseline social disparities and their changes over time during transition of economic crisis. In addition, 5-year interval ASDRs of each educational attainment subgroups were also contrasted with the highest education group (college or more) as a reference to decompose the each age groups' different contributions across four socioeconomic positions within the social disparities in life expectancies at age 40.

## Results

Table 1 features life expectancies by the level of education for men and women in five different time frames (1993, 1995, 2000, 2005 and 2010). Table 1 demonstrates that individuals with higher levels of education had longer life expectancies at age 40 in all three time points for both sexes. Also, the life expectancy at each level of education consistently increased over time. For instance, life expectancy of those with some elementary school experience or graduated increased from 28.9 in 1993, to 28.5 in 1995, to 29.5 in 2000, to 30.5 in 2005, and to 31.6 in 2010. Such an increase was consistently observed in all over the groups regardless of educational attainment and gender. It indicates that at least there was no mortality reversal in all four educational levels of Koreans during and post economic crisis.

**Table 1:** Life expectancy by education levels in Korea (1993, 1995, 2000, 2005, and 2010)

**MALE**	**1993**				**1995**				**2000**				**2005**				**2010**			
**Age**	**Elem**	**Middle**	**High**	**College**	**Elem**	**Middle**	**High**	**College**	**Elem**	**Middle**	**High**	**College**	**Elem**	**Middle**	**High**	**College**	**Elem**	**Middle**	**High**	**College**
40	28.9	31.1	34.5	36.9	28.5	32.3	33.9	38.0	29.5	33.9	35.2	39.2	30.5	35.2	37.5	41.1	31.6	36.3	39.1	42.8
45	25.8	27.0	29.9	32.1	25.8	28.2	29.4	33.2	26.7	30.0	30.7	34.4	27.8	31.5	32.9	36.3	28.8	33.1	34.6	38.1
50	22.4	23.0	25.5	27.5	22.6	24.2	25.0	28.6	23.6	25.9	26.3	29.7	25.0	27.7	28.5	31.6	26.1	29.6	30.2	33.4
55	19.4	19.3	21.5	23.2	19.5	20.4	20.8	24.2	20.4	22.0	22.0	25.2	22.0	23.6	24.1	27.0	23.1	25.8	25.9	28.8
60	16.2	15.8	17.6	19.2	16.4	16.8	16.9	20.1	17.3	18.2	18.1	21.0	18.6	19.6	20.0	22.6	19.9	21.8	21.6	24.3
65	12.9	12.6	13.9	15.3	13.2	13.4	13.3	16.1	14.1	14.7	14.3	17.0	15.5	15.9	16.1	18.5	16.5	17.9	17.6	20.0
70	10.0	9.6	10.8	12.0	10.3	10.3	10.2	12.8	11.0	11.4	10.9	13.3	12.5	12.6	12.6	14.6	13.4	14.3	13.9	16.0
75	8.0	7.3	8.5	9.5	8.1	8.0	7.8	10.0	8.3	8.6	8.1	10.1	9.5	9.6	9.4	11.2	10.6	11.1	10.7	12.4
80	5.6	5.1	6.2	6.7	5.8	5.7	5.5	7.4	6.1	6.2	5.9	7.4	7.1	7.0	6.8	8.4	8.0	8.6	7.8	9.4
85	4.5	4.6	4.8	5.2	4.6	4.2	4.0	5.6	4.5	4.6	4.5	5.5	5.2	5.1	5.2	6.4	6.1	6.7	5.8	7.2
**FEMALE**	**1993**				**1995**				**2000**				**2005**				**2010**			
**Age**	**Elem**	**Middle**	**High**	**College**	**Elem**	**Middle**	**High**	**College**	**Elem**	**Middle**	**High**	**College**	**Elem**	**Middle**	**High**	**College**	**Elem**	**Middle**	**High**	**College**
40	38.7	38.4	42.0	44.5	39.1	39.3	40.3	43.9	40.1	40.8	42.9	46.4	41.4	43.9	44.7	48.3	43.5	46.2	48.2	50.0
45	34.2	33.6	37.2	39.6	34.6	34.6	35.5	39.1	35.7	36.0	38.0	41.5	37.4	39.3	39.8	43.5	39.5	41.8	43.4	45.1
50	29.7	28.9	32.4	34.9	30.1	29.9	30.7	34.3	31.2	31.3	33.3	36.7	33.1	34.6	35.0	38.6	35.3	37.2	38.6	40.3
55	25.4	24.4	27.8	30.2	25.8	25.3	26.1	29.6	26.8	26.6	28.6	32.0	28.7	29.9	30.3	33.9	31.0	32.5	33.9	35.5
60	21.2	20.0	23.4	25.6	21.5	20.9	21.6	25.1	22.4	22.1	24.1	27.3	24.2	25.2	25.7	29.1	26.5	27.8	29.2	30.8
65	17.1	15.9	19.2	21.2	17.4	16.6	17.2	20.8	18.2	17.7	19.6	22.8	19.9	20.7	21.2	24.6	22.1	23.3	24.7	26.2
70	13.3	12.2	15.2	17.3	13.6	12.6	13.2	16.6	14.2	13.5	15.5	18.6	15.8	16.4	16.9	20.1	18.0	18.9	20.3	21.7
75	10.2	8.9	11.6	13.9	10.3	9.6	9.6	13.4	10.7	9.9	11.8	14.8	12.0	12.4	12.9	16.1	14.1	14.8	16.2	17.4
80	7.4	6.7	9.0	11.5	7.5	7.2	6.7	11.1	7.7	7.2	8.8	11.4	8.9	9.2	9.6	12.5	10.7	11.1	12.8	13.6
85	5.6	5.7	7.6	9.7	5.6	6.4	5.1	10.0	5.6	5.4	6.4	8.9	6.4	7.0	7.0	10.1	8.1	8.7	10.5	10.8

**Figure 1:**
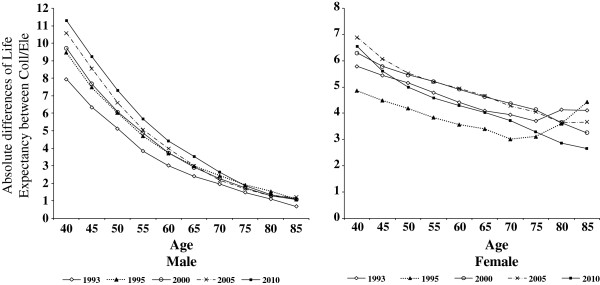
Absolute differences of life expectancy between college and elementary school of education, 1993–2010.

Table 2 is comprised of two panels. Panel (a) shows absolute differences in life expectancy at age 40 between those with some college or more education and other educational attainments; and panel (b) features rate ratios of life expectancy across educational attainments using the highest level as reference. Panel (a) shows the absolute gap in life expectancy between the highest and the lowest educated male Koreans increased from 9.47 in 1995 to 11.28 in 2010. The highest educated male Koreans outlived their lowest educated counterparts by 9.47 years at age 40 in 1995. The corresponding figures increased to 9.72 in 2000, to 10.59 in 2005, and to 11.28 in 2010. However, such a pattern was not observed in other levels of education. Indeed, the gap between the high school educated and college educated decreased from 4.09 in 1995 to 3.72 in 2010 (Figure 1). Among females, a gradual increase of absolute difference over time was found for the elementary educated and the high school educated compared to the college-or-more educated women. The absolute difference in life expectancy increased from 4.85 in 1995 to 6.54 in 2010 among the elementary educated, and from 3.64 in 1995 to 1.85 in 2010 among the high school educated compared to the college-or-more educated (Figure 1). Of interest are the rate ratios in panel (b). First, in males, relative discrepancy in life expectancy at age 40 across educational levels did not change or slightly decreased over time. However, in females, except for the middle school educated, the relative discrepancy worsened. Also interesting is the educational gradient of life expectancy as well as mortality was bigger among males than females, however the gap became notably worse from 1995 toward 2010 among females than males. For example, the social inequalities in life expectancy and mortality in females was smaller than males, however the social gradient become wider from 1995 to 2010 compared to males. Overall, the gradient of life expectancy at age 40 by educational attainment persisted during the period of economic crisis in Korea, although there were no life expectancy reversals even in the lowest educated groups. The absolute and relative discrepancies in life expectancy clearly indicate that the exacerbation was more pronounced in females than in males.

**Table 2:** Life expectancies disparities at age 40 years old by the level of educational attainments among Koreans (1993, 1995, 2000, 2005, and 2010)

**a. Absolute differences in life expectancy**			
**Male**	**Coll-Ele**	**Coll-Mid**	**Coll-High**
1993	7.95	5.74	2.38
1995	9.47	5.73	4.09
2000	9.72	5.23	3.92
2005	10.59	5.94	3.66
2010	11.28	6.56	3.72
**Female**			
1993	5.79	6.12	2.54
1995	4.85	4.58	3.64
2000	6.30	5.64	3.55
2005	6.89	4.46	3.68
2010	6.54	3.82	1.85
**b. Rate ratios across educational backgrounds**			
**Male**	**Ele/Coll**	**Mid/Coll**	**High/Coll**
1993	0.78	0.84	0.94
1995	0.75	0.85	0.89
2000	0.75	0.87	0.90
2005	0.74	0.86	0.91
2010	0.74	0.85	0.91
**Female**			
1993	0.87	0.86	0.94
1995	0.89	0.90	0.92
2000	0.86	0.88	0.92
2005	0.86	0.91	0.92
2010	0.87	0.92	0.96

**Figure 2:**
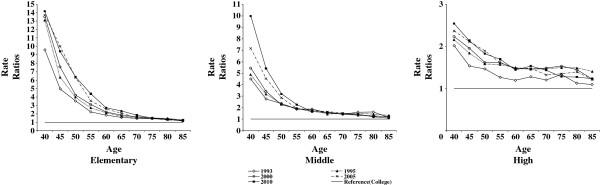
Changes in rate ratios of Age-Specific Death Rates (ASDR) among male Koreans, 1993–2010.

**Figure 3:**
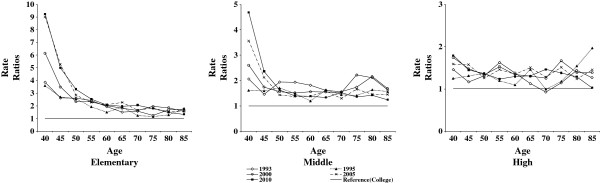
Changes in rate ratios of Age-Specific Death Rates (ASDR) among female Koreans, 1993–2010.

Life expectancy is affected by the mortality process of older age groups. The ASDR indicates the mortality experience of each age interval independent of other age intervals. Figures 2 and 3, respectively for males and females, feature rate ratios of ASDR of each educational level as reference to those received college education. For males, it is clear that the relative inequality in death rates increased over time among those in the working ages (40 to 59). On the other hand, little change was found over time for elderly elementary and middle school educated males (Figure 2). Relative inequality actually decreased among the high school educated elderly in 2010 as compared to previous years. The relative inequality in death rates worsened among elementary and middle school educated working aged adult females (about 40 to 50) while elderly females of these educational attainments and high school educated females (all age intervals) showed no patterned changes in their death rates over time (Figure 3). Overall, relative inequality in mortality increased among working-aged Korean males and lowly educated Korean females in their 40s as opposed to their elderly.

## Discussion

This study examined how the economic crisis in late 1990s and following neoliberal restructuring differently influenced the Korean people's life expectancies across different socioeconomic states. The intriguing result of this study is that the gap of social inequalities in life expectancy exists in Korea. The differences in life expectancy by education level appear to be more extreme than those previously reported based on Western societies. [13-22].

From the outset, we hypothesized that the gap of life expectancy at age 40 and ASDR across educational levels became widened as Korea went through the structural adjustment process imposed by IMF. Because the main thrust of the process was neo-liberalism, and deteriorating social inequality in health and mortality has been documented in a number of industrialized countries where neo-liberalism was expanded after their economic recession [1,2]. Based on the findings of this study, we are not able to simply determine that mortality disparity across levels of education among all Korean adults worsened over the study period. However, at least some pieces of evidence clearly indicate that the relative disparity in life expectancy at age 40 and ASDRs became worse among certain groups of Korean adults during and after the period of tremendous economic and social turbulence. They were the working aged Korean males and females (aged 40 to about 55), and Korean females were more victimized than males by the economic crisis or the introduction of neo-liberalism.

These results are quite comparable to outcomes of Russian crisis rooted from its political and economical turbulence during early 1990s. The Russian elementary-educated people showed tiny or almost no gaps versus the college-educated (below 3-year difference at age 20 in men; almost no difference in women) before crisis, but the elementary subgroups of both gender surprisingly lost 4-year life expectancy at 20 in a decade while the college ones gained about 6-year [23]. On the other hand, this study shows that Korean elementary-educated people had already tremendous gaps versus the college-educated even before the crisis (around 10- year life expectancy differences at age 40 in men and 4-year in women in 1995). However, the Korean elementary-educated still gained (3.1-year in men: 4.4-year in women, from 1995 to 2010) without reversal in their life expectancy at 40 in a decade although the college-educated acquired many more years (4.79 in men: 6.10 in women from 1995 to 2010). Korea exhibits much worse socioeconomic disparities in life expectancy between two subgroups nearly a decade after crisis compared to follow-up outcome in similar years after crisis in Russia. As mentioned above, a twofold mechanism by which neo-liberalism can increase health inequality can be considered: commodification of medical services and enlarged socioeconomic polarization. We believe the latter played a more important role than the former in widening mortality gap across educational groups among the working aged, particularly female Koreans. Of course, the commodification of once-publicly owned goods (such as energy, telecommunication, transportation and education) took place in full-scale right after the inception of IMF-proposed adjustment program [24,25]. However, at least the speed of health sector commodification was not accelerated being protected by the Korea National Health Insurance (NHI) system and the increased government spending on health services. Although the NHI often becomes the subject of criticisms regarding its limited coverage range and shows inequalities of health service utilization between different income groups, there is overall consensus that it has gradually reduced inequity in healthcare financing across income and occupational groups by expanding benefits (decreased premium and increased coverage) for Koreans of lower socioeconomic status [5]. Further, statistics show that governmental expenditure on health and social security has rather grown since 1997. For instance, the share of public health-related expenditure out of total government spending increased from 6.24% in 1995 to 12.42% in 2005 [11,26]. During this period, there was 665% increase in the Medical Aid budget [26], and according to the Korean government statistics [10], almost no change was found in the share of health care-related expenditure out of the total consumer expenditure since the economic crisis (from 4.6% in 1996 to 4.9% in 2005). Therefore, it is more likely that the increased socioeconomic polarization due to the expansion of neo-liberalism after the economic crisis was the major cause of increased mortality disparity discovered by this study.

Then why has the inequality in mortality increased only in working aged Koreans since the economic crisis? Labor market flexibility was one of the major conditions required by the IMF restructuring program. Under this condition and subsequent legalization of layoffs during corporate restructuring, companies, regardless of their size, began to actively utilize human resource policies that replaced regular workers with non-regular workers. The less educated or unskilled manual workers in their 40s and 50s were often the prime subject of layoffs or replacement. Before the economic crisis, employment in a company, regardless of the size of the company, meant lifetime employment in Korea. The concept of non-regular or part-time worker rarely existed in the country, and the most important factor that determined worker's salary was the seniority system. As the labor market became flexible and layoffs became legally supported, most companies began to downsize themselves by eliminating uncompetitive workers whose salary was relatively high but whose task was easily replaceable. The less educated or unskilled manual workers in their 40s and 50s who used to be protected by the lifetime employment inevitably became the victim of such transformation in the labor market. Increased insecurity in the job markets of the 40s and the 50s quickly worsened the share of non-regular jobs and a rapid surge in the size of the working-poor population, apart from the traditional poor. Then it gave rise to worsening inequalities and socioeconomic upheaval among all poor Koreans of working age [24,27]. Under the process of such economic restructuring, downward social mobilization was most prevalent among the 40s and the 50s [5,27,28], and, in turn, this phenomenon might have caused aggravated mortality inequality over time in these age groups as found in this study.

Another reason might be from inequalities in work. In Korea, education usually tends to determine future occupation. Lower educational level may act as a strong barrier to achieving a better occupation. Therefore the lower educated groups (less than high school level) tend to occupy manual labour jobs compared to the higher educated groups (higher than college level). After the economic crisis, work has been thoroughly intensified, based on the employers’ intention to increase productivity or, in other words, profits. Intensified work increases the physical demands placed on workers and can deteriorate the workers’ health, especially among the manual labour jobs. Therefore, the effects of socioeconomic disruption would have operated through work.

Our results showed that the adverse consequence of economic crisis regarding social differences in mortality by the levels of education was more pronounced for females than for males. It suggests that Korean women were victimized by the economic crisis to a much larger extent relative to their male counterparts. For centuries, Korea has been a male dominant society where husbands are the major bread winners and wives occupy more domestic roles. Since the economic crisis, women, particularly among the lower social classes, were driven out of the house and had to participate in the labor market due to a rapidly increased risk of unemployment and wage cutoffs of their husband. Because these women workers were unskilled, they mainly occupied jobs with very low wages and little or no job security [29,30]. Labor force participation itself, in general, could promote female health, given the positive relationship between work and health. However, when the low-educated women of extreme financial need entered the labor market, and had to continue their roles as housewives, it is more likely that the labor force participation harmed health rather than promoting it [29]. Thus, Korean women of low socioeconomic position had to experience the double jeopardy of household economy collapse and working in an unstable position under unhealthy working environment.

Our study has the following limitations. Firstly, as the data from the census and the national death files were not matched by individual person, misclassification of educational variable is likely in the census and national death data, which would be referred to as a numerator-denominator bias. Secondly, the use of education as a proxy for social class in this study might not be an accurate indicator for measuring true social class differentials. However, as the previous Korean studies showed that educational differences were more strongly related to the inequalities in health, therefore, the limitations of education would make the result less likely to be biased [31-36]. We also could not consider the changes of the educational system from 1995 to 2005. Education in Korea, especially college, had developed over the years. Therefore status of each education background may not be the same over the periods, but exactly who those individuals are and what specific risks they face cannot be determined with the available data. Thirdly, this study includes trends for the life expectancy measurement among Korean population only consider five different time periods (1993, 1995, 2000, 2005, and 2010). However, this data set supports our hypothesis by showing the typical changes among three different time periods: before (1993,1995), in the middle of economic crisis (2000), recovering period (2005,2010).

In conclusion, our study discovered that the harms were concentrated among low educated people, especially among working-aged population and women compared to elderly and men, during the late 1990s' economic decline and following neoliberal restructuring in Korea. These results may give lesson to current commodification process of medical services taken place in Korea, which have consisted of launching a government-level task force team to allow private hospitals modifying their legal status from non-profit to profit-making foundations as well as trying to make the private medical insurance as to supplement NHI. Given these processes of commodification of medical services, even though slow and gradual, it is very likely that social disparity in health such as life expectancy will be enlarged soon in Korea.

## Competing interests

The authors declare that they have no competing interests.

## Authors’ contributions

MS initiated the planning of the study, collected and analyzed data, and wrote the first draft. YC analyzed data, designed the entire analysis, and wrote the final manuscript. JO contributed to the study design and interpretation of the results. IK contributed to the initial study design and revision of the paper. JY acquired and analyzed data. SK was involved in the design of the study and the critical revision of manuscript. All authors read and agreed to the final draft of the manuscript.
